# The tetrapeptide core of the carrier peptide Xentry is cell-penetrating: novel activatable forms of Xentry

**DOI:** 10.1038/srep04900

**Published:** 2014-05-09

**Authors:** Kristopher Montrose, Yi Yang, Geoffrey W. Krissansen

**Affiliations:** 1 Department of Molecular Medicine & Pathology, Faculty of Medical and Health Sciences, University of Auckland, Auckland 1005, New Zealand

## Abstract

Here we describe a structure-function analysis of the cell-penetrating peptide Xentry derived from the X-protein of the hepatitis B virus. Remarkably, the tetrapeptide core LCLR retains the cell-penetrating ability of the parental peptide LCLRPVG, as either an L- or D-enantiomer. Substitution of the cysteine with leucine revealed that the cysteine is essential for activity. In contrast, the C-terminal arginine could be substituted in the L-isomer with lysine, histidine, glutamic acid, glutamine, and asparagine, though the resulting peptides displayed distinct cell-type-specific uptake. Substitution of the leucines in the D-isomer with other hydrophobic residues revealed that leucines are optimal for activity. Surprisingly, linear di- and tetra-peptide forms of Xentry are not cell-permeable. Protease-activatable forms of Xentry were created by fusing Xentry to itself via a protease-cleavable peptide, or by attaching a heparin mimic peptide to the N-terminus. These novel activatable forms of Xentry were only taken up by MCF-7 cells after cleavage by matrix metalloproteinase 9, and could be used to deliver drugs specifically to tumours.

Cell-penetrating peptides (CPPs) penetrate the plasma membrane, and are being utilized to deliver therapeutics to cells and tissues^1^,^2^,^3^,^4^,^5^. We recently reported on an entirely new class of CPP represented by the short peptide Xentry (LCLRPVG) derived from an N-terminal region of the X-protein of the hepatitis B virus^6^. The structure of Xentry is unlike that of other major classes of CPPs, which are generally 10 to 30 amino acid (aa) residues in length, and either arginine-rich, amphipathic and lysine-rich, or extensively hydrophobic^6^. Like several other CPPs, Xentry permeates adherent cells using syndecan-4 as a portal for entry, but is unique in being restricted from entering syndecan-deficient, non-adherent cells, such as resting blood cells^6^. This feature offers a therapeutic advantage as Xentry is not sequestered and diluted by blood cells when injected intravenously. Xentry has a predilection for uptake by epithelia. Thus, intravenous injection of Xentry either alone or conjugated to β-galactosidase led to its delivery to most tissues in mice, with the peptide becoming concentrated in epithelia overlying the bronchial airways and gastrointestinal tract^6^. Xentry is able to deliver an array of different cargo types to cells in an active form, including antibodies and siRNAs against B-raf, and large proteins such as β-galactosidase^6^. Xentry represents a new class of CPP with properties that are potentially advantageous for life science and therapeutic applications.

As with other CPPs, Xentry shows indiscriminate uptake by cells expressing the widely distributed syndecan-4, and potentially other members of this family. Tsien's group was the first to devise novel activatable CPPs (ACPPs) for selective delivery of drugs and imaging agents to tumours^7^,^8^,^9^. The cell-permeability of polycationic polyarginine-based CPPs is abrogated when they are fused to an inhibitory polyanionic stretch of negatively-charged glutamic acid residues due to the formation of an intramolecular hairpin^7^,^8^,^9^. Placement of a protease-cleavable linker peptide between the CPP and the inhibitory polyanionic sequence enabled the CPP to be activated by a protease which cleaved the linker, thereby releasing the CPP from the inhibitory polyanionic peptide^7^,^8^,^9^,^10^. The association of the CPP with cultured tumour cells increased 10-fold upon activation, and there was a 3-fold increase in uptake by tumours in mice, compared to contralateral normal tissue^7^. ACPP technology has been used to enhance the delivery of imaging agents, chemotherapeutic agents, and nanoparticles to tumours, and atherosclerotic plaques^10^,^11^,^12^,^13^,^14^,^15^,^16^,^17^, image apoptosis in the retina^18^, and has been developed to image diseases related to oxidative stress^19^.

The present structure-function study was designed to determine the features of Xentry that are essential for its cell-permeability. Here we define the minimal active motif, and determine amino acid substitutions that are tolerated and not tolerated. Two novel approaches have been devised to produce activatable forms of Xentry that could be used to selectively deliver drugs and imaging agents to tumours, and other diseased tissues.

## Results

### LCL(X) is the core motif which confers cell-penetrating ability

**Figure 1:**
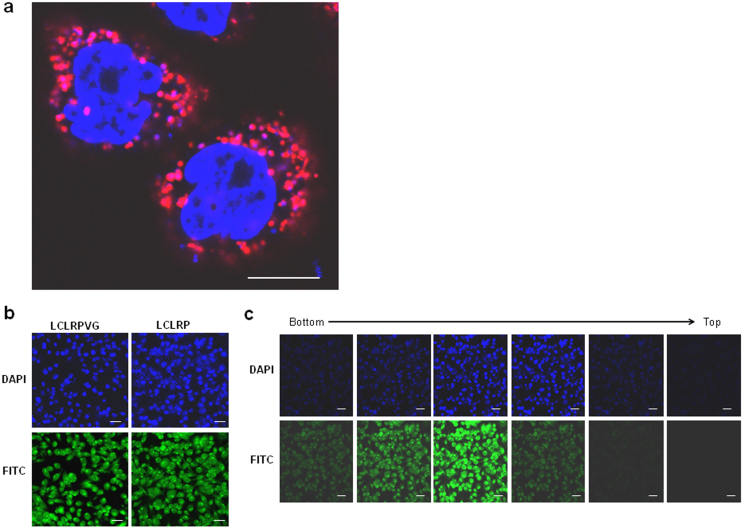
Confocal microscopy provides evidence that Xentry and its pentapeptide core are able to penetrate HepG2 cells. (a) High resolution confocal image of living HepG2 cells that have been incubated with TAMRA-labelled Xentry (10 μM) for 3 h. Cell nuclei were stained blue with hoescht stain. Images were taken at 63× magnification. Scale bar represents 10 μm. (b) Peptide LCLRP is taken up by HepG2 cells at an equivalent level to LCLRPVG. FITC-labelled peptides LCLRP and LCLRPVG were incubated with HepG2 cells for 3 h at a final concentration of 10 μM, and cell uptake visualised using a Leica TCS-SP2 confocal microscope. Cell nuclei were stained blue with DAPI. Images were taken at 25× magnification. (c) Confocal slicing of cells reveals that peptide LCLRP is taken up into the cytoplasm and nucleus. Multiple optical slices of the cell were taken from the bottom to the top of the cells. Images were taken at 25× magnification. Cell nuclei were stained blue with DAPI. Scale bar represents 50 μm.

**Figure 2:**
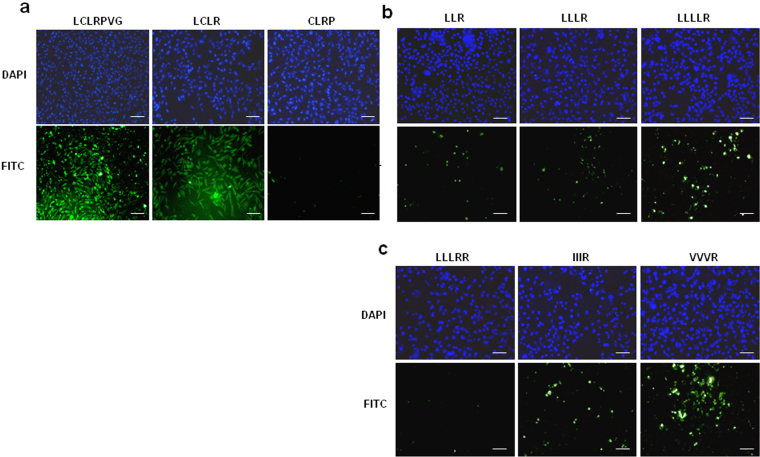
The tetrapeptide core of Xentry is able to penetrate HepG2 cells, whereas the truncated and modified peptides CLRP, LLR, LLLR, LLLLR, LLLRR, IIIR, and VVVR poorly penetrate HepG2 cells. FITC-labelled peptides LCLRPVG, LCLR, CLRP (a), LLR, LLLR, LLLLR (b), and LLLRR, IIIR, and VVVR (c) were incubated with HepG2 cells for 3 h at a final concentration of 10 μM. The cells were fixed and cell fluorescence recorded using a Nikon E600 fluorescence microscope. Cell nuclei were stained blue with DAPI. Images were taken at 20× magnification. Scale bar represents 50 μm.

High resolution confocal imaging of uptake of a D-isomeric form of TAMRA-labelled Xentry (lclrpvg) by living HepG2 cells revealed that Xentry is taken up into endosomes from which it is released as indicated by diffuse fluorescence in the cytoplasm surrounding the endosomes (Fig. 1a). Xentry did not concentrate at the cell-surface indicating that it is rapidly internalized. The FITC-labelled L-isomer of Xentry and the C-terminally truncated FITC-labelled peptide LCLRP were both taken up by HepG2 cells, as evidenced by confocal microscopy (Fig. 1b,c). The L-isomer of Xentry was N- and C-terminally truncated in order to identify the smallest cell-penetrating peptide. The LCLR peptide was readily taken up by HepG2 liver cancer cells, whereas the N-terminally truncated peptide CLRP was not cell-penetrating (Fig. 2a). Thus, amino acid (aa) residues LCLR are essential for conferring cell-penetrating ability. Three peptides containing a stretch of leucines followed by a single arginine residue (LLR, LLLR, LLLLR) were tested for uptake by the cell lines HepG2 (Fig. 2b), WM-266-4 (melanoma) and BT549 (epithelial breast carcinoma) (Supplementary Fig. 1) to determine whether cell-penetrating ability relates solely to N-terminal hydrophobicity. Surprisingly, the peptides poorly penetrated the three cell lines compared to the core LCLR peptide (Fig. 2a,b). Addition of an extra C-terminal arginine residue (LLLRR) failed to improve cell-penetrating ability, whereas substitution of the leucines with isoleucines and valines (IIIR, VVVR) improved cell uptake (Fig. 2c, Supplementary Fig. 1). Thus the cysteine at position 2 of LCLR appears to be critical for cell-penetration.

**Figure 3:**
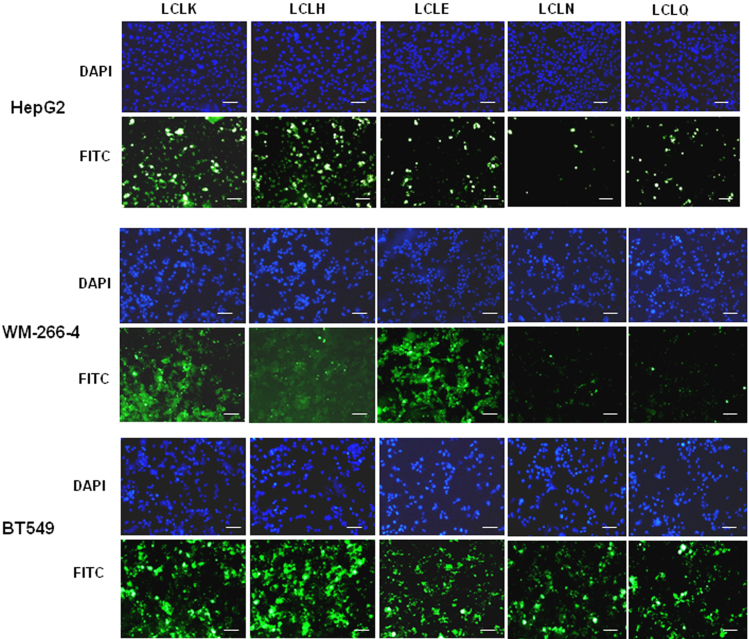
Substitution of the 4^th^ residue of the tetrapeptide core alters cell-specificity. FITC-labelled peptides LCLK, LCLH, LCLE, LCLN, and LCLQ were incubated with HepG2, WM-266-4, and BT549 cells for 3 h at a final concentration of 10 μM, and cell uptake visualized using a Nikon E600 fluorescence microscope. Cell nuclei were stained blue with DAPI. Images were taken at 20× magnification. Scale bar represents 50 μm.

The importance of the C-terminal arginine residue was tested by its substitution with conservative (lysine and histidine) and non-conservative (glutamic acid) residues, giving the L-isomeric peptides LCLK, LCLH, and LCLE, respectively. The LCLK and LCLH peptide variants were cell-penetrating as predicted, but surprisingly the LCLE peptide also penetrated the HepG2, WM-266-4, and BT549 cell lines (Fig. 3). LCLK was taken up well by all three cell lines as might be expected. However, the other variant peptides displayed cell-type restricted permeability (Fig. 3). Thus LCLH was readily taken up by HepG2 and BT549, but less well by WM-266-4 cells, whereas LCLE was readily taken up by WM-266-4 cells and BT549 cells, but less well by HepG2 cells (Fig. 3). Substitution of the C-terminal arginine residue with the non-charged polar residue glutamine (LCLQ) maintained cell-permeability for the BT549 cell line, but not the WM-266-4 cell line, and did not improve uptake by HepG2 cells. Substitution of the C-terminal arginine residue with the non-charged polar residue asparagine (LCLN) gave similar results. Thus the C-terminal residue (LCLX) controls the degree of cell-penetration, and dictates cell-specific penetrating ability.

**Figure 4:**
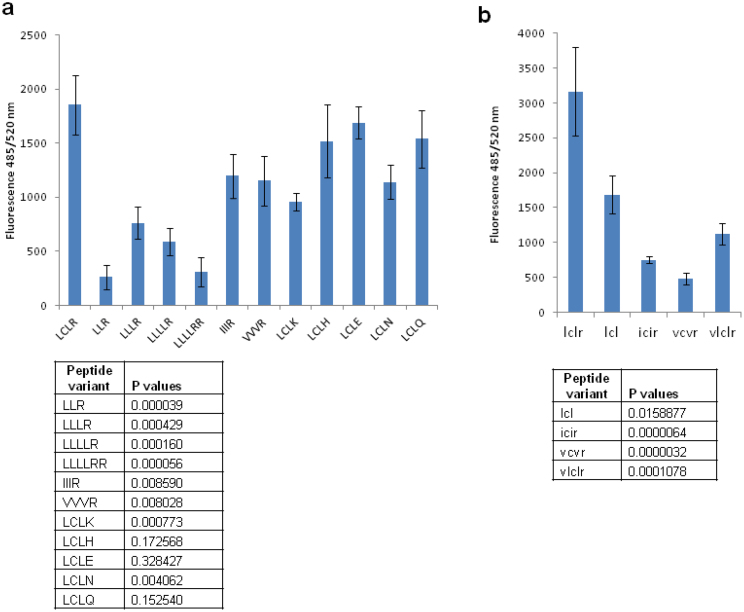
Measurement of uptake of Xentry variant peptides by HepG2 cells. HepG2 cells seeded into 96-well plates were incubated with L-isomeric (a), and D-isomeric (b) peptides at a final concentration of 10 μM in quadruplicate wells. The cells were incubated for 3 h, fixed with 4% formaldehyde, and fluorescence measured using a fluorescent plate reader at an excitation wavelength of 485 nm and emission wavelength of 520 nm. The fluorescence of cells was plotted as the mean ± SD (n = 4). The statistical significance of the differences in cell fluorescence compared to cells treated with the Xentry tetrapeptide is shown in the tables beneath each graph.

Uptake of the variant Xentry peptides by HepG2 cells was quantified by measuring the overall cell fluorescence in wells following peptide treatment. This assay confirmed that omission of the cysteine greatly reduced the cell-penetrating ability of Xentry (Fig. 4a). The cell-penetrating abilities of the LCLH, LCLE, and LCLQ peptides were not significantly different from that of LCLR, whereas the LCLK and LCLN peptides displayed reduced activity.

### D-isomeric truncated forms of Xentry are cell-penetrating and stable in serum

We had previously reported that a D-isomeric form of Xentry was cell-penetrating, and more stable in serum than the L-isomer^6^. FITC- and TAMRA-labelled D-isomers of Xentry were readily taken up by HepG2 cells at concentrations as low as 5 and 0.75 μM, respectively (Supplementary Fig. 2a). The truncated D-isomeric peptide lclrp was readily taken up by HepG2 and/or WM-266-4 cells in the absence and presence of serum (Supplementary Fig. 2b), and could be preincubated in serum for 4 h without apparent loss of activity, whereas the L-isomer was rendered non-functional after 1 h (Supplementary Fig. 2c). The D-isomeric form of the core motif lclr was cell-permeable at all concentrations (10 to 100 μM) of peptide tested (Supplementary Fig. 2d).

### Leucines are optimal for cell-penetration

**Figure 5:**
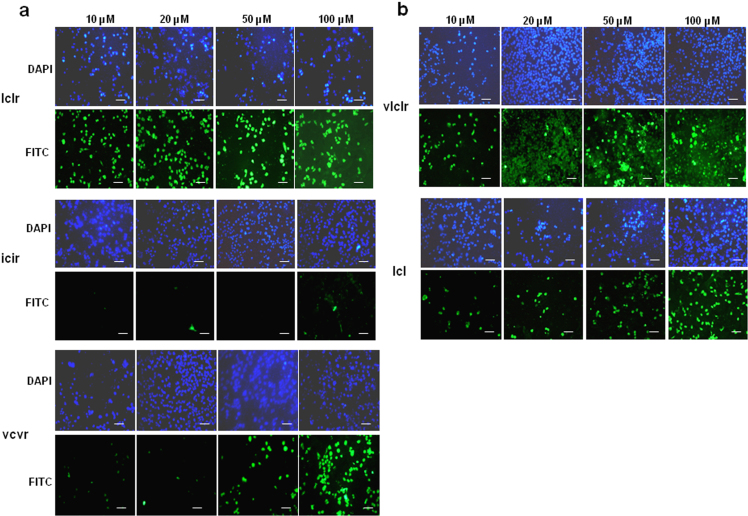
Leucine residues are optimal for the cell-penetrating ability of Xentry. D-isomeric FITC-labelled peptides lclr, icir, vcvr (a), and vlclr and lcl (b) were incubated with HepG2 cells for 3 h at concentrations of 10, 20, 50 and 100 μM. Cell uptake was visualized using a Nikon E600 fluorescence microscope. Cell nuclei were stained blue with DAPI. Images were taken at 20× magnification. Scale bar represents 50 μm.

Conservative substitution of the two leucines flanking the cysteine residue with either isoleucine or valine greatly reduced the cell-penetrating ability of the D-isomeric lclr peptide. Thus, the icir peptide showed poor uptake by HepG2 cells, and the vcvr peptide was only taken up effectively at high concentrations (Fig. 5a). Addition of a valine residue to the N-terminus to give the peptide vlclr, as in the wild-type X-protein sequence, had no major effect as cell uptake was comparable to that of lclr at the higher concentrations of peptide (Fig. 5b). Removal of the C-terminal arginine to give the simple tripeptide lcl was predicted to inactivate the peptide, but surprisingly the peptide at high concentrations remained moderately cell-penetrating for a subpopulation of cells (Fig. 5b). Thus, the tripeptide lcl appears to be the core cell-penetrating motif, where addition of the C-terminal arginine residue enhances cell-penetration. Substitution of the leucines for other hydrophobic residues causes decreased cell-penetration. The results for peptide concentrations of 10 μM were confirmed by quantifying the overall cell fluorescence in wells (Fig. 4b). Table 1 summarizes the ability of the different Xentry variants to penetrate the HepG2, WM-266-45, and BT549 cell lines, as determined by visual analysis.

### Secondary structure of Xentry

**Figure 6:**
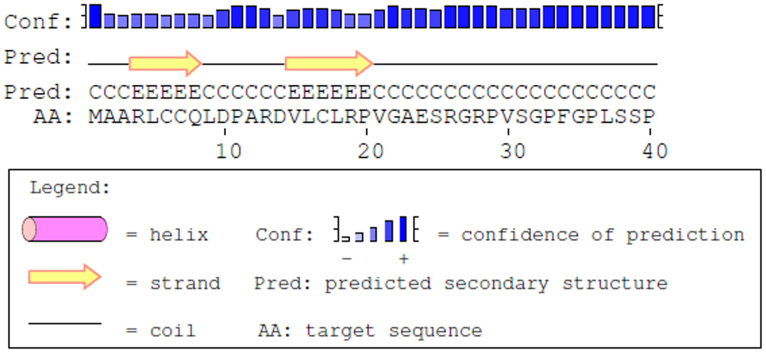
The tetrapeptide core of Xentry lies within a β-strand. According to the PSIPRED protein structure prediction server, the sequence encompassing the N-terminal 40 aa residues of the X-protein contains 2 β-strands, with the tetrapeptide core of Xentry being embedded in the second β-strand.

According to the PSIPRED protein structure prediction server (http://bioinf.cs.ucl.ac.uk/psipred/)^20^, the sequence encompassing the N-terminal 40 aa residues of the X-protein contains two β-strands, with the tetrapeptide core of Xentry being embedded in the second β-strand (Fig. 6). The smallest Xentry-containing X-protein peptide (DVLCLRPVGA) accepted by the server confirmed that Xentry forms a β-strand (data not shown).

### Truncated and D-isomeric forms of Xentry are unable to penetrate resting lymphocytes

**Figure 7:**
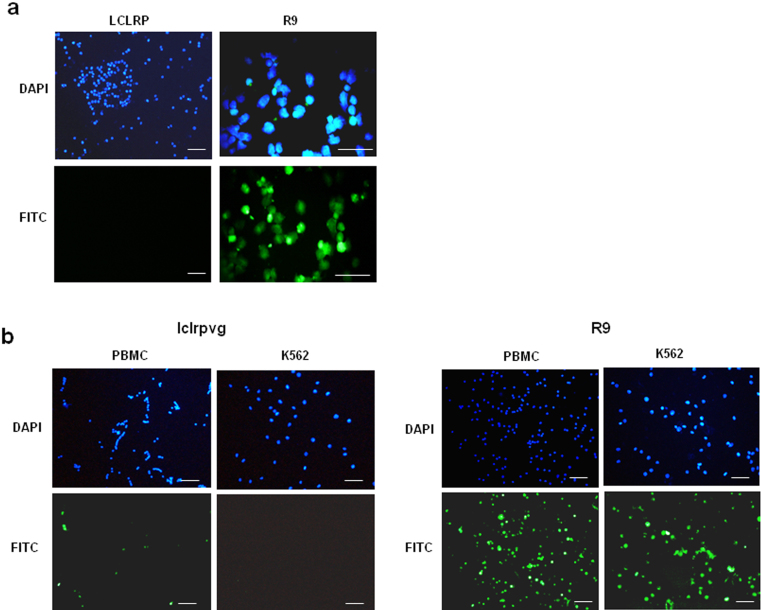
Truncated and D-isomeric forms of Xentry are unable to penetrate resting lymphocytes. (a) FITC-labelled peptides LCLRP and R9 were incubated with TK-1 cells in suspension for 3 h at a final concentration of 10 μM. (b) The FITC-labelled peptides lclrpvg and R9 were incubated with PBMCs isolated from a buffy coat, and with K-562 cells in suspension for 3 h at a final concentration of 10 μM. Cell uptake visualized using a Nikon E600 fluorescence microscope. Cell nuclei were stained blue with DAPI. Images were taken at 20× magnification, except for R9 image in a) which was taken at 40×. Scale bar represents 50 μm.

A key feature of Xentry is its inability to penetrate resting blood cells, and non-adherent leukemic cell lines which are deficient in glycosylated syndecans^6^. The truncated peptide LCLRP (10 μM) was also unable to penetrate the TK-1 thymic lymphoma cell line, whereas in contrast a polyarginine (R9) peptide was readily taken up by these cells (Fig. 7a). Further, the D-isomeric form of Xentry (lclrpvg) was unable to penetrate unactivated peripheral blood mononuclear cells, and the K562 erythroleukemia cell line (Fig. 7b). In contrast, a polyarginine (R9) peptide was readily taken up by both cell types.

### Xentry is non-toxic to cells

**Figure 8:**
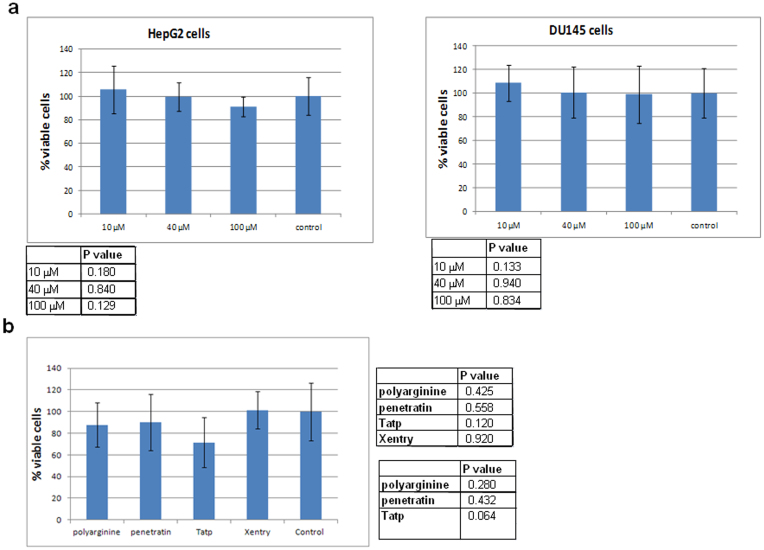
Xentry is non-toxic to HepG2 and DU145 cells. (a) HepG2 and DU145 cells were incubated for 24 h with 0, 10, 40, and 100 μM of FITC-labelled D-isomeric Xentry (lclrpvg) for 24 h, and cell viability measured using the WST-1 assay. The mean percentage of viable cells was plotted ± SD. The statistical significance of the differences in cell viability compared to cells that were not treated with Xentry is shown in the tables beneath each graph. (b) HepG2 cells were incubated for 24 h with biotin- labelled Xentry (LCLRPVGGGRRRQQQQQQRRR), penetratin (GRKKRRQRRRPPQGGRRRQQQQQQRRR), polyarginine (RRRRRRRRRQQQQQQRRR) and Tatp (RQIKIWFQNRRMKWKKGGRRRQQQQQQRRR) peptides at final concentrations of 10 μM. Cell viability was measured using the WST-1 assay. The mean percentage of viable cells was plotted ± SD. Untreated HepG2 cells were included as controls. The statistical significance of the differences in cell viability after treatment with each of the CPPs compared to untreated control cells is shown in the top right-hand table. The statistical significance of the differences in cell viability after treatment with Xentry compared to the other CPPs is shown in the bottom right-hand table.

A FITC-labelled D-isomeric form of Xentry (lclrpvg) at concentrations of 10, 40 and 100 μM did not significantly (p > 0.05) affect the cell viability of HepG2 cells, and the DU145 prostate cancer cell line (Fig. 8a). Approximately 90% of HepG2 cells and 98% of DU145 cells were still viable even at the highest peptide concentration of 100 μM. The potential toxicity of biotinylated L-isomeric Xentry fused to a stretch of 6 glutamine residues surrounded by arginine residues (LCLRPVGGGRRRQQQQQQRRR), which we have used as a transglutamination site for conjugating protein cargoes^6^, was compared with the toxicities of the polyarginine (R9), Tatp, and penetratin also containing the transglutamination site. All four CPPs (10 μM) had no significant affect on the viability of HepG2 cells, and there was no significant difference in the toxicity of Xentry compared to the other CPPs (Fig. 8b). Tatp inhibited cell viability the most amongst the four CPPs, but the affect did not reach significance, whereas Xentry had the least effect on the viability of the cells.

### Linear polymers of Xentry are not able to penetrate cells

**Figure 9:**
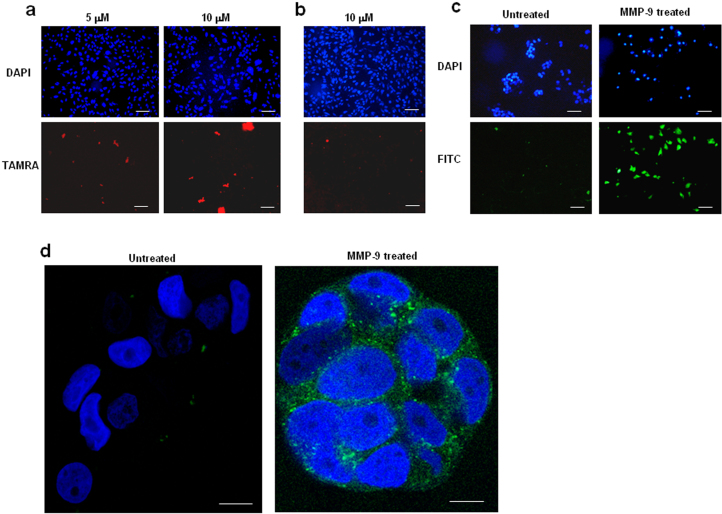
Linear multivalent and heparin mimic-conjugated forms of Xentry are not cell-penetrating, but have utility as protease-activatable peptides. A TAMRA-labelled divalent D-isomer (lclrpvggggggggggggggglclrpvg) (a), and a divalent L-isomer (LCLRPVGGGGGGGGGGGGGGGLCLRPVG) (b) of Xentry are unable to penetrate HepG2 cells. The peptides were incubated for 3 h with HepG2 cells at concentrations of 5 and/or 10 μM, as indicated. Cells were fixed and cell fluorescence recorded using a Nikon E600 fluorescence microscope. Cell nuclei were stained blue with DAPI. Images were taken at 20× magnification. Scale bar represents 50 μm. The small amount of red fluorescence is not cell-associated. (c, d) Activatable forms of Xentry. (c) The ACPP lclrpvGGGGPLGLAGGlclrpvgk-FITC peptide at a final concentration of 10 μM was left untreated or incubated for 3 h with 0.2 μg of activated MMP-9, and then incubated with MCF-7 cells for 3 h. The cells were fixed and fluorescence was visualized and recorded using a Nikon E600 fluorescence microscope. Cell nuclei were stained blue with DAPI. Images were taken at 20× magnification. Scale bar represents 50 μm. (d) The ACPP GSY(sulfated)DY(sulfated)GGGGPLGLAGGlclrpvgk-FITC peptide at a final concentration of 10 μM was left untreated or incubated for 3 h with 0.2 μg of activated MMP-9, and then incubated with MCF-7 cells for 3 h. The cells were fixed and fluorescence was visualized and recorded using a Zeiss LSM 710 inverted confocal microscope. Cell nuclei were stained blue with DAPI. Images were taken at 63× magnification. Scale bar represents 10 μm.

Divalent ((lclrpvggggggggggggggglclrpvg; Fig. 9a) and tetravalent (lclrpvglclrpvglclrpvglclrpvg; data not shown) TAMRA-labelled D-isomeric forms of Xentry at concentrations of 5 and/or 10 μM were surprisingly completely unable to penetrate HepG2 cells. The tetravalent D-isomer appeared to become stuck to the outside of the cell (**data not shown**). There was also no uptake of the L-isomeric form of the divalent peptide (Fig. 9b).

### Activatable Xentry peptides

CPPs have been devised so that they are only cell-penetrating after activation by a protease^7^. ACPPs have potential application in targeting the delivery of therapeutic cargoes to tumours as they are selectively activated at the tumour site by proteases secreted by the tumour. Here, two entirely different activatable forms of Xentry were developed. The above results (Fig. 9a,b) had shown that linear polyvalent forms of Xentry are not cell-penetrating, suggesting that for whatever reason the addition of a second Xentry motif in close proximity on the same peptide blocks cell uptake. This finding provided the basis of a novel approach to create an activatable form of Xentry. A FITC-labelled peptide lclrpvGGGGPLGLAGGlclrpvgk-FITC was synthesized, consisting of two D-isomeric Xentry motifs separated by a linker peptide containing an L-isomeric MMP-9 cleavage site. Pretreatment of the peptide with matrix-metalloproteinase 9 (MMP9) led to uptake of the digested peptide by MCF-7 breast cancer cells, which secrete only a low level of MMP-9^17^, whereas the untreated peptide was not cell-permeable (Fig. 9c).

We had previously shown that heparin prevents the uptake of Xentry by HepG2 cells, in accord with the fact that Xentry is taken up by the heparin sulphate proteoglycan syndecan-4^6^. Heparan sulfate is one the most negatively charged molecules found in nature, as it bears a high number of negatively-charged sulfate groups^21^. The peptide GGGGSY(sulfated)DY(sulfated)GGGG (designated SPa) containing two sulfate groups was devised by Kim and Kiick as a heparin mimetic^22^, and was considered here to be a candidate inhibitor of Xentry. It was fused N-terminally to Xentry via a linker peptide that contained an MMP-9 cleavage motif. A C-terminal fluoresceinated lysine was added to Xentry, giving the peptide GSY(sulfated)DY(sulfated)GGGGPLGLAGGlclrpvgk-FITC. The uncleaved peptide was not taken up by MCF-7 cells (Fig. 9d). Once cleaved by MMP-9, the activated peptide was readily taken up by the cells, as confirmed by confocal microscopy (Fig. 9d). It is hypothesized that the highly negatively-charged sulfate groups within the blocking peptide render the peptide unable to interact appropriately with cell-surface HSPGs on syndecan-4. The release of the sulphated blocking peptide by MMP-9 cleavage allows uptake of the freed Xentry peptide into the cell.

## Discussion

Xentry represents a unique class of CPP in that it is very short, has only a single charged arginine residue, bears no resemblance to any previously described CPP, and does not share with other CPPs the ability to enter cells by a non-endocytic route^6^. The results of the present study demonstrate that the tetrapeptide core of Xentry remarkably retains the cell-penetrating ability of the parental peptide. Even the tripeptide lcl penetrated a subpopulation of HepG2 cells. The cell-penetrating ability of Xentry appears to be predominantly sequence-specific, unlike that of polyarginine-based CPPs. The hydrophobic head of Xentry comprises a cysteine residue flanked by two leucine residues. The cysteine residue appears to be obligatory for cell function, and the leucines cannot be conservatively substituted with other hydrophobic residues without some loss of function. The cysteine residue in Xentry may play a key role in intermolecular disulphide bonding, by mediating the bonding of Xentry to itself, or to cell-surface thiols. Thiol-containing peptides have been reported to cross-react with cell-surface thiols, becoming either trapped in the plasma membrane or internalized^23^. Leucine, isoleucine and valine are each hydrophobic branched-chain aa. It is not known why leucine is optimal for the cell-penetrating ability of Xentry. There is no preference for leucines over other hydrophobic aa in other CPPs^24^. Amino acid position 4 of Xentry appears to be a “wobble” position in that restriction of the nature of the amino acid at this position is somewhat relaxed. Thus, conservative (his, lys), and nonconservative (glu, asn, gln) amino acids can be accommodated. However, whilst LCLR and LCLK were taken up by all cells examined, substitution of arginine with other amino acids resulted in cell type-specific uptake. Thus the aa at position 4 of the tetrapeptide enhances cell-permeability and dictates cell-specificity. Exactly how the “wobble aa” at position 4 dictates cell-specificity is unknown, but presumably it plays a key role in the interaction of Xentry with cell-surface HSPGs, including members of the syndecan family. It may therefore be possible to preferentially target Xentry to particular tissue types by simply changing the amino acid at position 4.

The tetrapeptide core of Xentry forms part of a β-strand, which is interesting as β-strands participate in protein-protein interactions. Substitution of the cysteine for leucine, and substitution of LCL for III and VVV, does not disrupt the predicted β-strand structure (data not shown). Neither does substitution of arginine for glutamic acid, or glutamine. Thus inhibition of the function of Xentry by substitution with the above aa does not reflect changes to secondary structure, but rather changes to the primary sequence, and potentially the tertiary structure. Substitution of arginine for asparagine truncated the β-strand by 2 aa (data not shown), yet cell uptake by LCLN was similar to that of LCLQ. Xentry was non-toxic to cells, and truncated and D-isomeric forms retain the essential functions of Xentry, including its inability to penetrate resting leukocytes.

Conjugation of multiple copies of the cationic CPP Tatp, and the amphipathic CPPs penetratin and TP10 to linear scaffolds, creating branched structures, has been reported to enhance their cell permeability^25^,^26^. Further, dimerization of penetratin via addition of a C-terminal cysteine enhanced its ability to deliver DNA to cells^27^. Unexpectedly, synthetic linear divalent and tetravalent forms of Xentry were completely non-functional. There are several potential explanations. Placement of two Xentry motifs adjacent to one another may cause steric hindrance at binding sites on the cell-surface. The adjacent motifs may interact physically with one another eg via the cysteines which may form an intra-chain disulfide bond that could inactivate the obligatory cysteine. Alternatively, multivalency may increase the occurrence of inter-chain disulfide bonding, and thus the likelihood of peptide aggregation. Whatever the explanation, the phenomenon offered the opportunity to devise a novel activatable form of Xentry. An ACPP was created by placement of a protease-cleavable linker peptide between juxtaposed Xentry peptides. Cleavage of the peptide with MMP9 would be expected to release two active Xentry monomers. Accordingly, the C-terminal Xentry, which was fluorescently labelled, was selectively taken up by cells following protease cleavage.

Our previous study had suggested that Xentry enters cells exclusively via an energy-dependent endocytic process, and unlike other CPPs it cannot enter cells passively^6^,^28^,^29^. As shown in the present study, Xentry is initially taken up and concentrated into endosomes, similar to the Tat peptide^30^. Uptake of Xentry by cells is via the clathrin pathway, and is dependent on HSPG-decorated syndecan-4^6^. HSPGs and syndecans are known for their ability to internalize physiological extracellular ligands, viruses, bacteria and basic peptides^31^. Syndecans are reported to internalize cargoes via clathrin-independent pathways^31^, but this is not always the case as exemplified by the ability of R-Spondin to induce syndecan-4-dependent, clathrin-mediated, endocytosis^32^. Syndecan-4 has been reported to bind and transport the cationic CPPs penetratin, octaarginine and TATp into the cells via its heparan sulfate chains^33^. The fact that Xentry cannot enter HSPG-deficient K562 erythroleukemic cells, and resting T and B cells is further evidence that it requires HSPGs for cell uptake^6^. In accord, we previously showed that heparin blocks the cell uptake of Xentry^6^. Native heparin was initially considered as a blocking moiety for attachment to Xentry, but it is a highly sulfated glycosaminoglycan polymer with a molecular weight ranging from 3 to 30 kDa^34^, and would have been difficult to work with. Instead, a truncated form of the short heparin mimetic peptide GGGGSY (sulfated)DY(sulfated)GGGG (SPa) devised by Kim and Kiick^22^ was employed. SPa was shown to have the highest affinity of binding for heparin binding peptides and VEGF165 amongst a panel of heparin mimetic peptides^22^. Xentry fused to SPa via a protease-cleavable linker peptide was only taken up by MCF-7 cells after pretreatment with MMP9.

In summary, the tetrapeptide core LCLR of Xentry is cell-penetrating, where the aa at position 4 enhances the cell-penetrating ability of the minimal motif LCL, and dictates cell-specificity. Surprisingly, synthetic linear polymers of Xentry are not cell-penetrating, but offer the opportunity to develop novel ACPPs for tissue-specific delivery. Novel activatable forms of Xentry fused to heparin mimetics offer a new class of ACPPs that could also be used for tissue-specific delivery. Conceivably, the protease-cleavable linker peptide in the above ACPPs could be replaced with other labile linkers to target not just tumours, but an array of other diseased tissue types, as exemplified by a recent hydrogen peroxide-activated CPP incorporating a boronic acid-containing cleavable linker which will have utility in imaging tissues subjected to oxidative stress^19^.

Xentry was initially isolated from the 154 amino acid residue X-protein, which is just one of six proteins encoded by the hepatitis B virus that chronically infects 400 million people worldwide^35^. The X-protein contains domains involved in oligomerization, transactivation, and apoptosis. The potential role of Xentry, if any, within the X-protein is unknown. The native X-protein is not cell-penetrating, indicating that the Xentry peptide would need to be unmasked by proteolysis if it were to facilitate cell uptake of X-protein peptides.

Further research development of Xentry will require understanding the role of the cysteine residue, how internalization of Xentry is triggered, the mechanism by which Xentry is released from endosomes to enable this process to be enhanced to improve drug delivery, and whether Xentry interacts directly or indirectly with syndecan-4 and other syndecans. Xentry has a predilection for concentrating in epithelia^6^, hence the clinical translation of Xentry peptides could focus on Xentry-mediated delivery of drugs to diseased epithelia, including carcinomas where the activatable forms of Xentry described in this report will have utility. The finding that Xentry is taken up selectively by activated lymphocytes and not by resting lymphocytes^6^ suggests Xentry could be used to deliver drugs to activated immune cells in the treatment of inflammatory conditions, thereby sparing the remainder of the immune system.

## Methods

### Cells and peptides

The human HepG2 (liver cancer), WM-266-4 (melanoma), MCF-7 (breast cancer), BT549 (breast cancer), DU145 (prostate cancer), K562 (erythroleukemia), and mouse TK-1 (thymic lymphoma) cell lines were obtained from the American Type Culture Collection (ATCC). The HepG2, WM-266-4, MCF-7 and DU145 cell lines were propagated in full MEM media at 37°C and 5% CO2, while the BT549, K562, and TK-1 cells were propagated in full RPMI 1640 media at 37°C and 5% CO2. All peptides were synthesized by Peptide 2.0 Inc., Chantilly, VA.

### Assay for testing the cell-penetrating ability of Xentry peptides

Cells were seeded into 8-well chamber slides at 1 × 10^5^ cells per well in media with 10% FCS and PSG, and cultured overnight at 37°C and 5% CO2. The cells were then washed thrice with serum-free media. L-isomeric peptides were diluted in 500 μl of media without FCS, whereas D-isomeric peptides were diluted in the same media with FCS, and each added to cells at the indicated final concentrations. The cells were incubated for 3 h at 37°C and 5% CO2, washed with PBS, fixed with 4% formaldehyde in PBS for 30 min, and again washed with PBS. A drop of Prolong Gold anti-fade reagent with DAPI (Life technologies) was added to each sample. The cells were then examined by microscopy by using a Nikon E600 fluorescence microscope. For further examination, cells were examined by confocal microscopy using a Leica TCS-SP2 confocal microscope. For high resolution imaging of uptake of TAMRA-labelled Xentry by living HepG2 cells, the cells were treated as above, but after incubation for 3 h they were washed with PBS and 500 μl of full MEM medium was added back to the wells. The cells were reincubated at 37°C and 5% CO2 for approximately 30 min, then the living cells were examined with a Zeiss LSM 710 inverted confocal microscope.

### Assay for quantifying the ability of Xentry variant peptides to penetrate HepG2 cells

HepG2 cells were seeded into 96-well plates at 2.5 × 10^4^ cells per well in 200 μl of MEM media containing 10% FCS and PSG, and cultured overnight at 37°C and 5% CO2. The cells were washed thrice with media without FCS, and peptides diluted in 100 μl of MEM media without FCS were added to the cells at a final concentration of 10 μM in quadruplicate wells. The cells were incubated for 3 h at 37°C and 5% CO2, then washed thoroughly with PBS, fixed with 4% formaldehyde for 30 min, and fluorescence measured using a fluorescent plate reader at an excitation wavelength of 485 nm and emission wavelength of 520 nm. The fluorescence of cells was plotted as the mean ± SD (n = 4).

### Testing the ability of activatable forms of Xentry to penetrate MCF7 cells

Recombinant human MMP-9 expressed in mouse NSO cells (Sigma-Aldrich) was activated prior to use with 2.5 mM aminophenyl mercural acetate (APMA). The activatable Xentry peptides (10 μM) were incubated for 3 h at 37°C in the presence or absence of 0.4 μg/ml of activated MMP-9. The peptides were added to overnight cultures of 1 × 10^5^ MCF7 cells in 8-well chamber slides, and the plates incubated for 3 h at 37°C and 5% CO2. The cells were fixed with 4% formaldehyde for 30 min, and a drop of Prolong Gold antifade reagent with DAPI (Life technologies) was added to each well. The cells were examined and photographed using a Nikon E600 fluorescence microscope, or a Zeiss LSM 710 inverted confocal microscope.

### Assay for uptake of Xentry peptides by peripheral blood mononuclear cells

Venous blood was fractionated on Ficoll-paque Plus (GE Healthcare), and the buffy coat containing peripheral blood mononuclear cells was collected. The blood cells (5 × 10^5^) were incubated in an 8-well chamber slide for 3 h with 10 μM of peptide in RPMI media containing 10% FCS to give an adherent monocyte population and non-adherent lymphocyte population. The non-adherent lymphocytes were collected, and cytospun onto a slide, and examined by microscopy using a Nikon E600 fluorescence microscope.

### WST-1 cell proliferation assay

Cells were seeded into 96-well plates at 2.5 × 10^4^ cells per well in MEM media with 10% FCS and PSG, and cultured overnight at 37°C and 5% CO_2_. The cells were washed and peptide diluted in MEM media without FCS was added at the indicated final concentrations in triplicate wells. The cells were incubated for 3 h at 37°C and 5% CO2, after which the media was replaced with full MEM media containing FCS, and the cells incubated overnight at 37°C and 5% CO2. WST-1 cell proliferation reagent (Clontech) was added to the wells, followed by incubation for 90 min at 37°C and 5% CO2. The absorbance of each well was measured on a plate reader at 440 nm, with background levels measured at 650 nm.

### Statistical analysis

Results were expressed as mean values and standard deviation, and a Student's t-test was used for evaluating statistical significance for comparison between groups. A value less than 0.05 (p < 0.05) indicated statistical significance.

## Author Contributions

G.W.K. conceived and directed the research. K.M. conducted the majority of the experiments. Y.Y. provided technical help, and conducted some experiments. G.W.K. and K.M. analyzed the data. G.W.K. and K.M. wrote the manuscript. All authors commented on the manuscript.

## Supplementary Material

Supplementary Information Supplementary figures

## Figures and Tables

**Table 1:** Cell-penetrating ability of Xentry and variants for different cell lines

Peptide	Uptake by HepG2 cells	Uptake by WM-266-4 cells	Uptake by BT-549cells
**L-isomers**			
LCLRPVG	+++	+++	+++
LCLRP	+++	+++	+++
LCLR	+++	NT	NT
CLRP	-	NT	NT
LLR	-	-	++
LLLR	-	-	-
LLLLR	+	-	+
LLLRR	-	-	-
IIIR	++	-	++
VVVR	++	-	-
LCLK	+++	+++	+++
LCLH	+++	++	+++
LCLE	++	+++	+++
LCLN	+	+	+++
LCLQ	++	+	+++
**D-isomers**			
lclrpvg	+++	+++	NT
lclrp	+++	+++	NT
lclr	+++	NT	NT
lcl	+	NT	NT
icir	-	NT	NT
vcvr	+	NT	NT
vlclr	++	NT	NT

Peptide uptake by cells was scored visually according to the system: (-) no uptake; (+) weak uptake; (++) moderate uptake; (+++) strong uptake; (NT) not tested.
