# Supplementary material for: The tetrapeptide core of the carrier peptide Xentry is cell-penetrating: novel activatable forms of Xentry
Source: Sci Rep. 2014 May 9;4:4900. doi: 10.1038/srep04900 (PMC4014984; doi:10.1038/srep04900)
Supplement: Supplementary Information — Supplementary figures [file srep04900-s1.pdf]

The tetrapeptide core of the carrier peptide Xentry is cell-penetrating: novel activatable forms of Xentry

Kristopher Montrose, Yi Yang, and Geoffrey W. Krissansen

[1]Department of Molecular Medicine & Pathology, Faculty of Medical and Health Sciences, University of Auckland, Auckland 1005, New Zealand.

Supplementary Figure 1

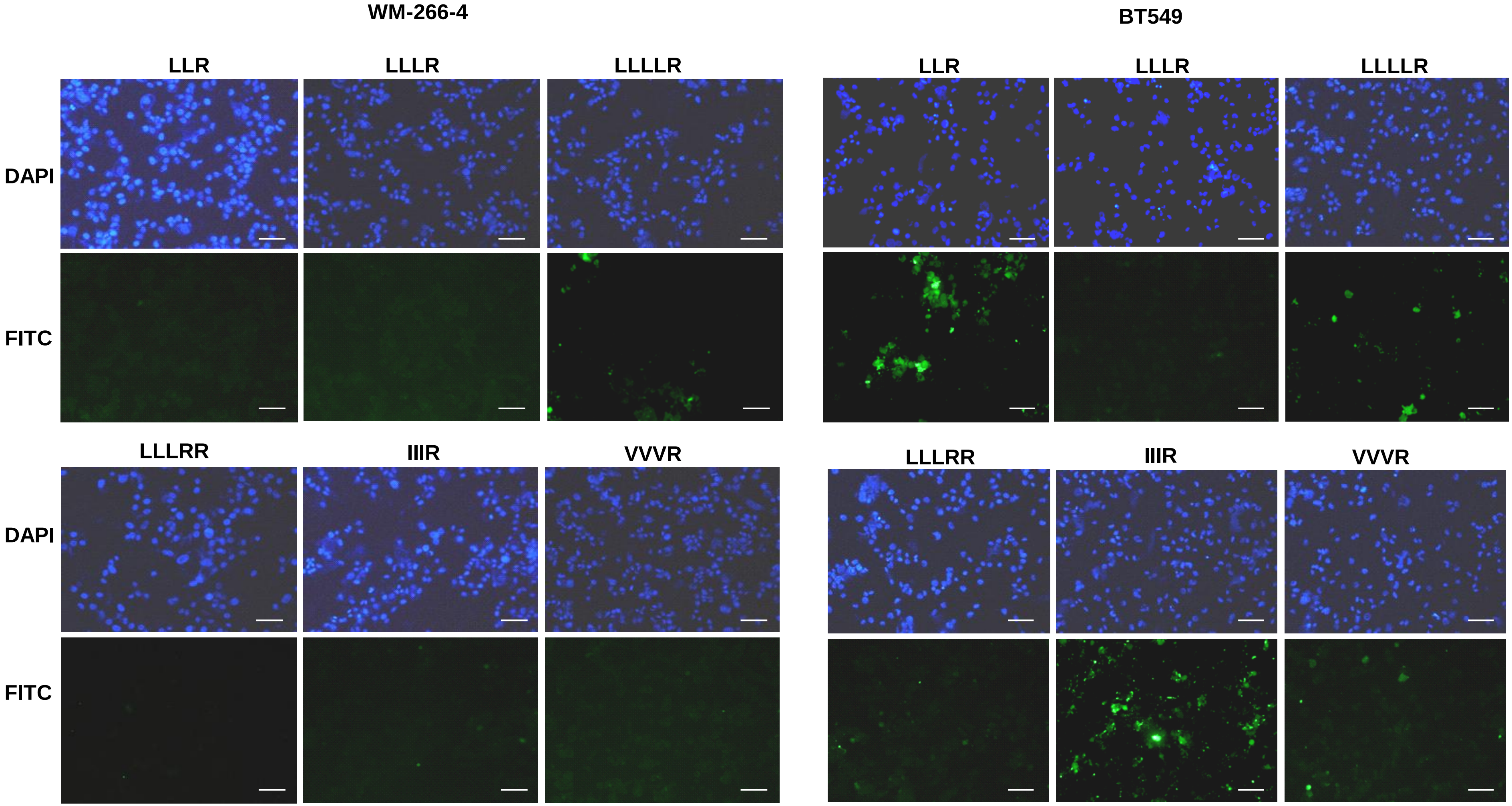

Supplementary Figure 1. Peptides LLR, LLLR, LLLLR, LLLRR, IIIR, and VVVR are poorly cell-permeable for WM-266-4 and BT549 cells. FITC-labelled peptides LLR, LLLR, LLLLR, LLLRR, IIIR, and VVVR were incubated with WM-266-4 and BT549 cells for 3 h at a final concentration of 10 μM. The cells were fixed and cell fluorescence recorded using a Nikon E600 fluorescence microscope. Cell nuclei were stained blue with DAPI. Images were taken at 20x magnification. Scale bar represents 50 μm.

Supplementary Figure 2a

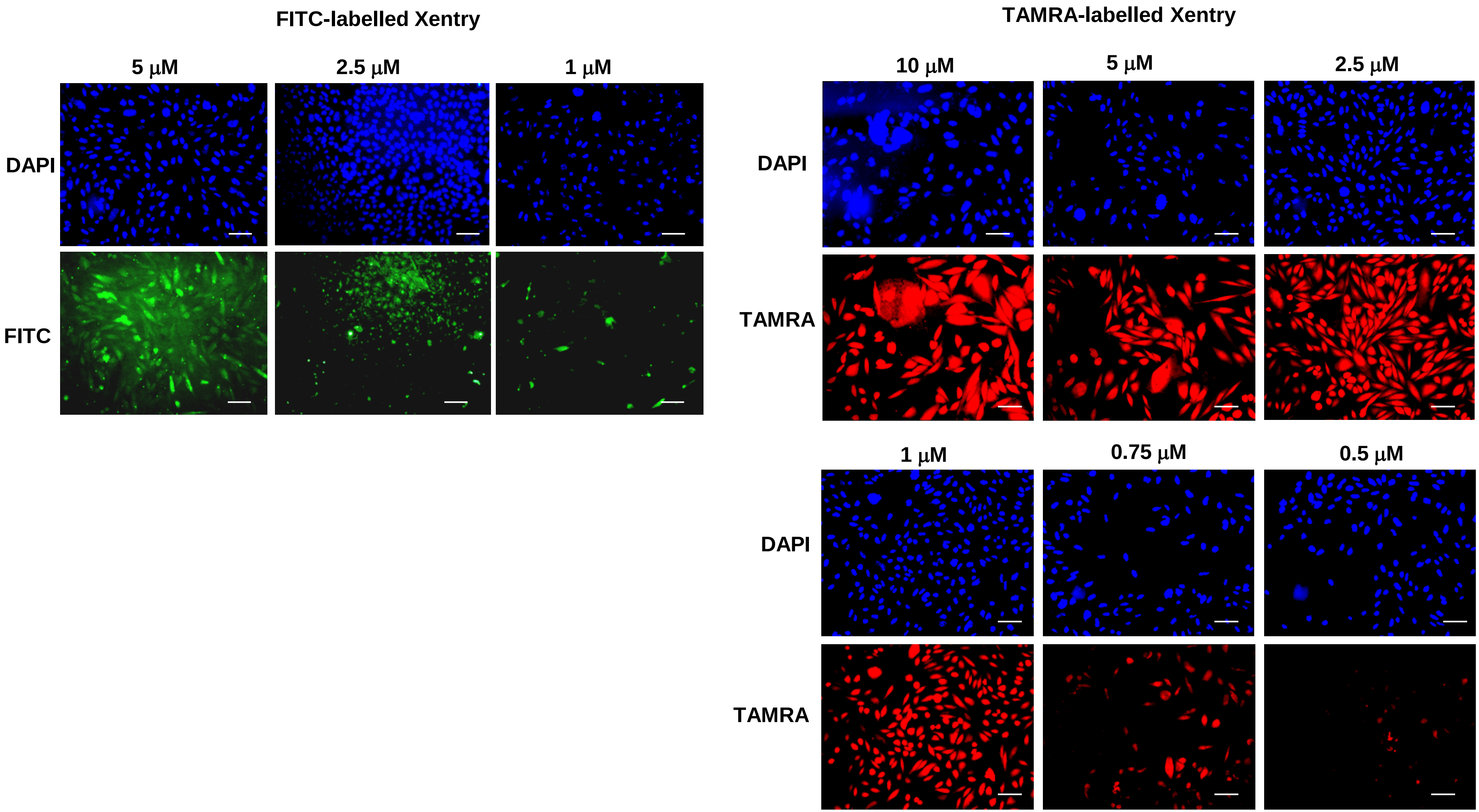

Supplementary Figure 2a. Uptake of D-isomeric forms of Xentry by HepG2 cells is concentration-dependent. FITC-labelled and TAMRA-labelled D-isomers of the peptide lclrpvg were incubated for 3 h with HepG2 cells at decreasing concentrations, as indicated. Cells were fixed and cell fluorescence recorded using a Nikon E600 fluorescence microscope. Cell nuclei were stained blue with DAPI. Images were taken at 20x magnification. Scale bar represents 50 μm.

Supplementary Figure 2b

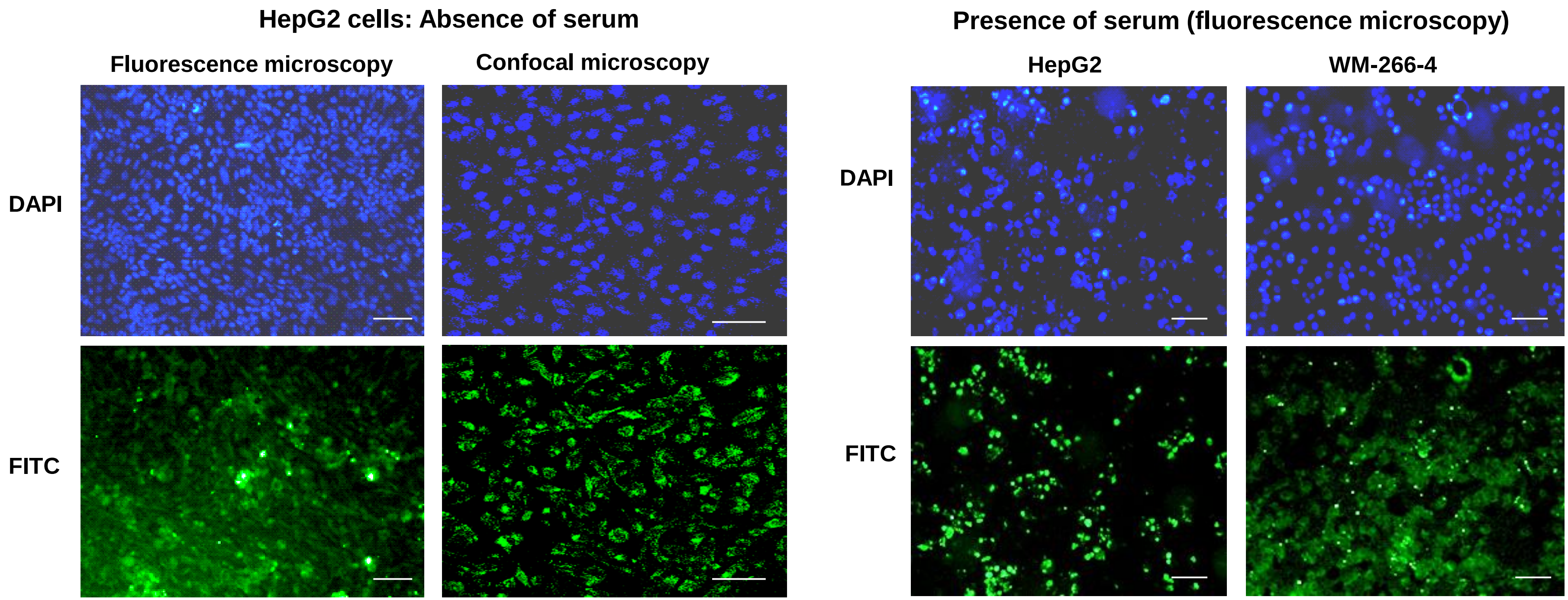

Supplementary Figure 2b. The short D-isomeric peptide lclrp is able to penetrate HepG2 and WM-266-4 cells in the presence or absence of serum. The FITC-labelled peptide lclrp at a final concentration of 10 μM was incubated for 3 h with HepG2 cells in non-serum-containing media, or with HepG2 and WM-266-4 cells in serum-containing media, as indicated. The cells were fixed and cell fluorescence recorded using both a Nikon E600 fluorescence microscope and a Leica TCS-SP2 confocal microscope, as indicated. Cell nuclei were stained blue with DAPI. Images were taken at 20x magnification for fluorescence microscopy, and at 25x magnification for confocal microscopy. Scale bar represents 50 μm.

Supplementary Figure 2c

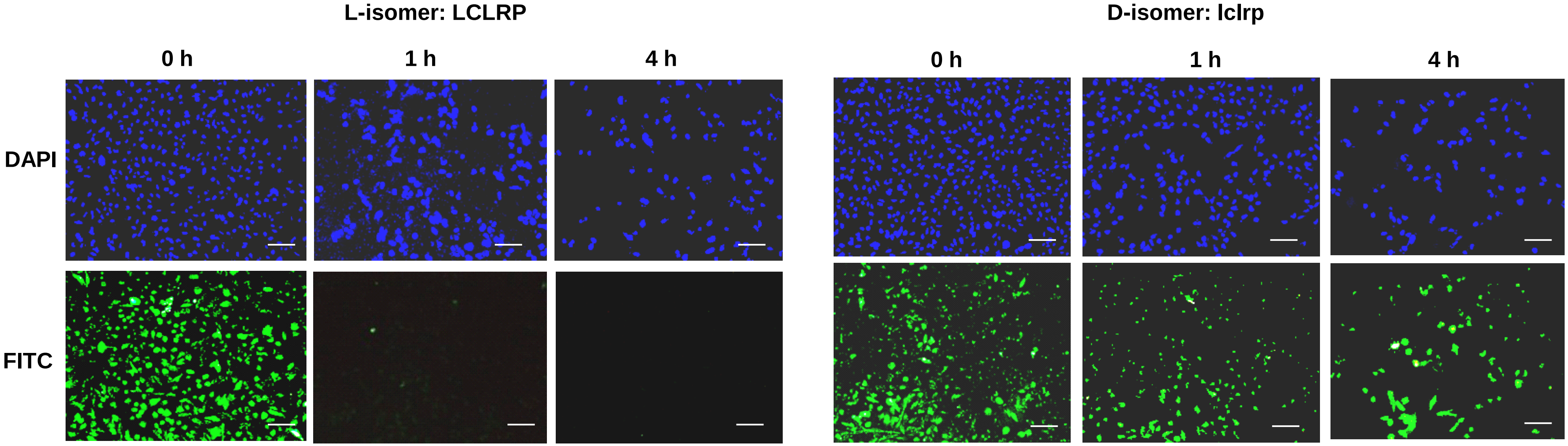

Supplementary Figure 2c. The D-isomeric peptide lclrp retains its cell-penetrating ability after pre-incubation in the presence of serum, whereas the L-isomeric peptide LCLRP is rendered inactive. The FITC-labelled peptides lclrp and LCLRP were pre-incubated at a final concentration of 10 μM in media containing 10% FCS for 0, 1, and 4 h, and then incubated with HepG2 cells for 3 h. The cells were fixed and cell fluorescence recorded using a Nikon E600 fluorescence microscope. Cell nuclei were stained blue with DAPI. Images were taken at 20x magnification. Scale bar represents 50 μm.

Supplementary Figure 2d

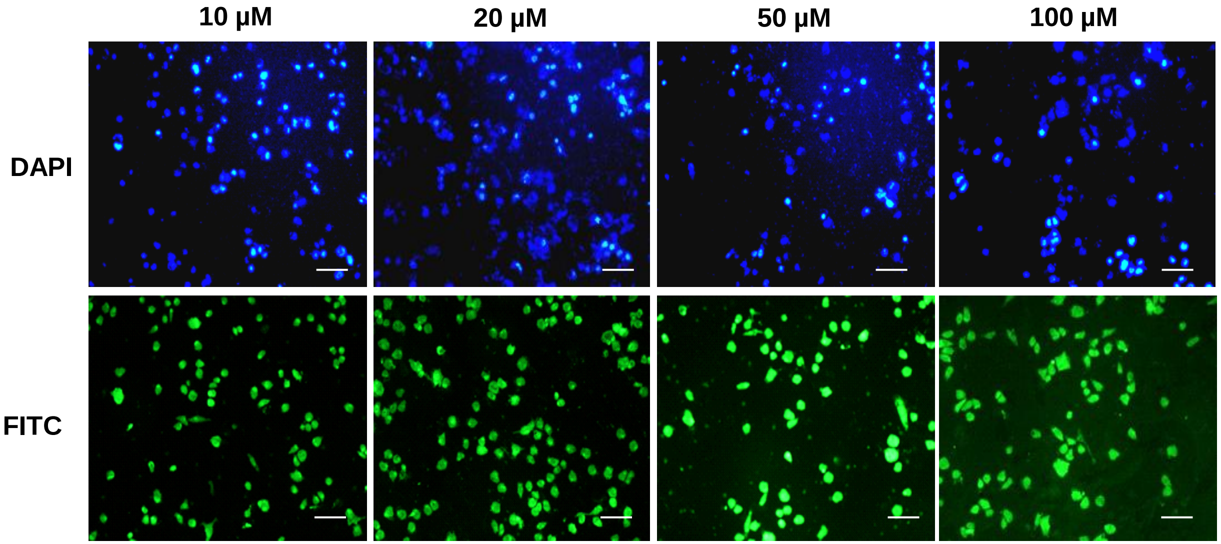

Supplementary Figure 2d. The D-isomeric peptide lclr permeates HepG2 cells. The FITC-labelled peptide lclr was incubated with HepG2 cells for 3 h at concentrations of 10, 20, 50 and 100 µM. The cells were fixed and cell fluorescence recorded using a Nikon E600 fluorescence microscope. Cell nuclei were stained blue with DAPI. Images were taken at 20x magnification. Scale bar represents 50 µm.
